# P13, the EMBL macromolecular crystallography beamline at the low-emittance PETRA III ring for high- and low-energy phasing with variable beam focusing

**DOI:** 10.1107/S1600577516016465

**Published:** 2017-01-01

**Authors:** Michele Cianci, Gleb Bourenkov, Guillaume Pompidor, Ivars Karpics, Johanna Kallio, Isabel Bento, Manfred Roessle, Florent Cipriani, Stefan Fiedler, Thomas R. Schneider

**Affiliations:** a Hamburg Unit c/o DESY, European Molecular Biology Laboratory (EMBL), Notkestrasse 85, 22603 Hamburg, Germany; b Fachhochschule Lübeck, Fachbereich Angewandte Naturwissenschaften, Mönkhofer Weg 239, 23562 Lübeck, Germany; c European Molecular Biology Laboratory, Institut Laue-Langevin, BP 181, 6 rue Jules Horowitz, 38042 Grenoble Cedex 9, France

**Keywords:** phasing, sulfur SAD, variable beam, low-energy data collection, 2θ-detector arm, softer X-rays, tunability

## Abstract

The macromolecular crystallography P13 beamline is part of the European Molecular Biology Laboratory Integrated Facility for Structural Biology at PETRA III (DESY, Hamburg, Germany) and has been in user operation since mid-2013. P13 is tunable across the energy range from 4 to 17.5 keV to support crystallographic data acquisition exploiting a wide range of elemental absorption edges for experimental phase determination. An adaptive Kirk­patrick–Baez focusing system provides an X-ray beam with a high photon flux and tunable focus size to adapt to diverse experimental situations. Data collections at energies as low as 4 keV (λ = 3.1 Å) are possible due to a beamline design minimizing background and maximizing photon flux particularly at low energy (up to 10^11^ photons s^−1^ at 4 keV), a custom calibration of the PILATUS 6M-F detector for use at low energies, and the availability of a helium path. At high energies, the high photon flux (5.4 × 10^11^ photons s^−1^ at 17.5 keV) combined with a large area detector mounted on a 2θ arm allows data collection to sub-atomic resolution (0.55 Å). A peak flux of about 8.0 × 10^12^ photons s^−1^ is reached at 11 keV. Automated sample mounting is available by means of the robotic sample changer ‘MARVIN’ with a dewar capacity of 160 samples. In close proximity to the beamline, laboratories have been set up for sample preparation and characterization; a laboratory specifically equipped for on-site heavy atom derivatization with a library of more than 150 compounds is available to beamline users.

## Abstract 2

The P13 macromolecular crystallography beamline, based on the low-emittance source PETRA III, enables X-ray diffraction experiments on macromolecular crystals over a wide wavelength range (0.7–3.1 Å). The beam has a variable focus size and a small divergence enabling data collection on micrometre-sized crystals.

## 1. Introduction   

In 2009, DESY completed the conversion of the 2.3 km-long PETRA ring, previously used as an injector for high-energy physics experiments on HERA, into a dedicated synchrotron radiation source with an emittance of 1.1 nm rad (Balewski *et al.*, 2004 ▸). On PETRA III, three beamlines for structural biology have been designed and constructed by EMBL Hamburg: P12 for small-angle X-ray scattering on solutions of biological macromolecules (SAXS) in sector 8, and P13 and P14 for macromolecular crystallography (MX) in sector 9. The beamlines are embedded in the ‘EMBL Integrated Facility for Structural Biology’ that provides instruments and support for the characterization, preparation and crystallization of samples (Boivin *et al.*, 2016 ▸) for subsequent use in SAXS and/or MX experiments.

P13 was designed to exploit the unique properties of PETRA III in terms of low emittance and high energy for addressing challenging crystal structure determinations. For phasing applications, tunability across a wide range of energies (4–17.5 keV) was implemented to exploit many different elemental absorption edges for MAD (multi-wavelength anomalous diffraction) or SAD (single-wavelength anomalous diffraction) phasing. An optical configuration with a beam demagnification of 1:12 horizontally and 1:15 vertically was chosen to maintain a small beam divergence while achieving a 20–30 µm spot size to be able to extract high-quality diffraction data from small crystals with large unit cells. To provide optimum conditions for different types of crystals, adaptive X-ray mirrors, exchangeable apertures and finely adjustable beam absorbers have been installed to tailor the beam properties rapidly to match the properties of a crystal mounted on the diffractometer. In the design and during the commissioning of the beamline, particular attention was paid to enabling data collections at energies lower than 6 keV to enhance the anomalous signal measurable from sulfur and/or phospho­rus atoms while maintaining high photon fluxes into small beam cross sections. Data collection at energies above 6 keV is performed in air, while for energies between 4 and 6 keV a helium cone is used to reduce air-scatter. Other beamlines capable of reaching low energies have chosen other approaches. While the I23 beamline at Diamond (UK) (Wagner *et al.*, 2016 ▸) features a complete *in vacuum* setup and a wavelength range from 1.5 to 4 Å (8–3 keV), the BL-1A beamline at the Photon Factory (Tsukuba, Japan) (Liebschner *et al.*, 2016 ▸) offers a full *in helium* setup with limited tunability extending over the wavelength ranges 0.95–1.1 Å (12.5–11.5 keV) and 2.7–3.3 Å (4.6–3.7 keV).

**Figure 1:**
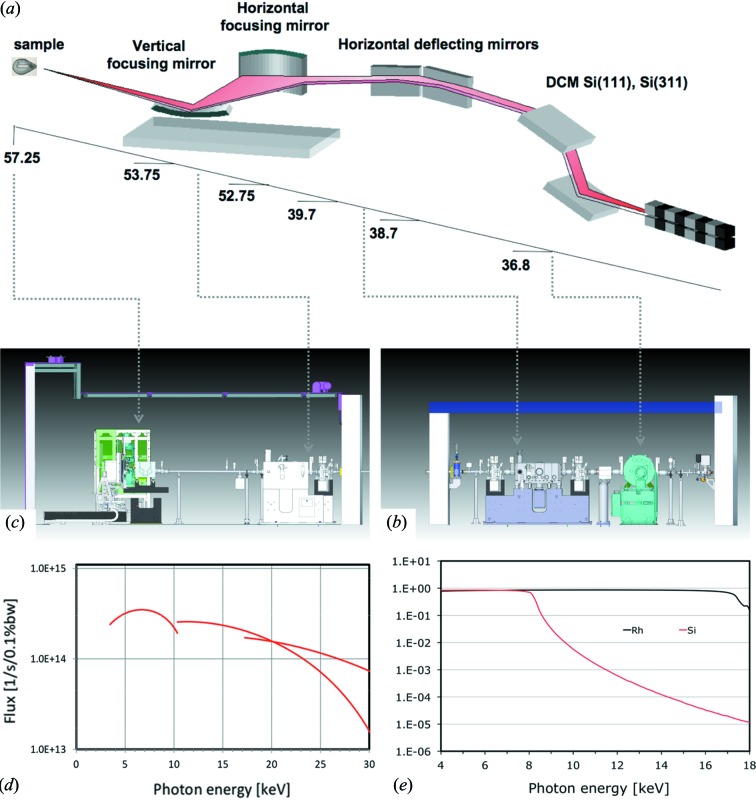
P13 beamline. (*a*) Optical layout; assembly drawing of (*b*) the optics hutch, (*c*) the experimental hutch, (*d*) predicted flux of the 2 m U29 undulator through a 1 mm × 1 mm aperture at 40 m distance from the source at the PETRA III storage ring (DESY, Hamburg), (*e*) reflectivity of photon beam *versus* energy through the double horizontal deflecting mirror for Rh coating (black line) and uncoated silica (red line).

The co-location of beamlines P13 and P14 on a pair of canted undulators in a relative short and narrow sector of the PETRA III synchrotron posed a number of technical challenges that needed to be addressed in design and construction. The design of beamline P13 began in 2007, with general user operation commencing in 2013 (for a more detailed timeline see Table S1 ▸ of the supporting information). The overall layout of the beamline is shown in Figs. 1(*a*)–1(*c*) ▸; details can be found in Table 1 ▸.

## 2. Beamline overview   

### 2.1. Storage ring and insertion device   

PETRA III is a storage ring with a circumference of 2304 m operated at an energy of 6.084 GeV with a horizontal emittance of 1.1 nm rad (Franz *et al.*, 2007 ▸). The ring current is injected in top-up mode with various bunch-filling patterns (40, 60, 240, 480, 960 bunch modes) up to a current of 100 mA in normal operation. In the 5 m-long high-β straight section of sector 9 of the PETRA III ring a pair of undulators with a canting angle of 5 mrad produces two independent X-ray beams for the P13 and P14 beamlines. The U29-2 2 m-long undulator (Barthelmess *et al.*, 2008 ▸) deployed in P13 produces an X-ray beam with dimensions (RMS) of 140 µm (H) × 5.6 µm (V) and divergence (RMS) of 7.9 µrad (H) × 4.2 µrad (V) at 10 keV (Balewski *et al.*, 2004 ▸; Franz *et al.*, 2007 ▸). The energy ranges for the different harmonics are shown in Fig. 1(*d*) ▸.

### 2.2. Monochromator   

A customized version of the high-heat-load FMB-OXFORD (Oxford, UK) double-crystal monochromator (DCM) has been installed as the first optical element at 36.8 m distance from the source (Fig. 1*a* ▸). The DCM contains two interchangeable pairs of crystals: while the Si(111) crystals are used for standard applications over the entire energy range with Δ*E*/*E* < 2 × 10^−4^, the Si(311) crystals can be used for applications requiring high-energy resolution (Δ*E*/*E* < 5 × 10^−5^) over a limited energy range (6–17 keV). The monochromator is indirectly cooled by a closed-loop liquid-nitro­gen (LN2) cryostat. The first crystal is clamped between side-cooling blocks through which the cryogen is flowing. The cooling blocks of the second crystal are coupled by copper braids to the cooling blocks of the first crystal. After initial exposure of the cold monochromator to the full white beam, thermal equilibrium of the second crystal with temperature fluctuations of less than 0.1°C is reached in about 45 min. The vibrational stability of the double-crystal set has been iteratively improved to currently better than 85 nrad r.m.s. during static operation.

### 2.3. Horizontal deflecting mirrors   

Following the design used at GM/CA-CAT at the Advanced Photon Source (Fischetti *et al.*, 2005 ▸, 2007 ▸), we introduced two horizontally deflecting mirrors (HDMs) at 38.7 m and 39.7 m from the source, respectively, each providing a fixed 3.8 mrad incident angle resulting in a total horizontal deflection of the beam of 15.2 mrad (Figs. 1*a*, 1*b* ▸), with a corresponding horizontal separation between the two beams at the P13 sample position of 548 mm (Fig. 3). Specifications of the two mirrors, manufactured by SESO (Aix-en-Provence, France), are reported in Table 1 ▸. Bruker ASC (Bergisch-Gladbach, Germany) supplied the HDM mirror assembly. High-resolution slope error measurements of the mirror surfaces were carried out at the Helmholtz-Zentrum-Berlin (BESSY-II, Institut für Nanometer Optik und Technologie) with the nanometer optical component measuring machine (NOM), according to the protocol reported by Siewert *et al.* (2012 ▸) for the characterization of the P13 KB mirrors. A Rh-coated region is used for operation in the energy range 6–17.5 keV. The uncoated silica region is used at energies below 6 keV and facilitates the suppression of high-energy harmonics (Fig. 1*e* ▸). The reflectivity of each mirror is above 70% between 4 keV and 17 keV. For energies above 17 keV, the reflectivity drops sharply to less than 20%.

### 2.4. Focusing mirrors   

**Figure 2:**
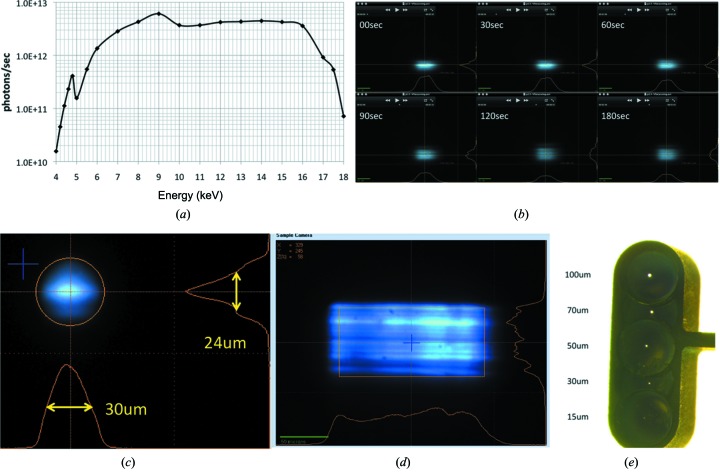
Beam size and photon flux. (*a*) Total photon flux as a function of energy at the sample position for fully focused beam; (*b*) time course of beam profile during the vertical defocusing procedure from 24 µm to 70 µm over a period of 180 s; (*c*) beam profile of the fully focused beam 30 µm (H) × 24 µm (V) at the sample position, determined with the built-in scintillator of MD2; (*d*) defocused beam of 150 µm (H) × 70 µm (V) size; (*e*) MD2 penta-aperture with aperture sizes indicated.

A pair of multi-segmented piezo-electrically adaptable mirrors (Signorato *et al.*, 1998 ▸), manufactured by SESO (Aix-en-Provence, France), commonly referred to as bimorph mirrors, in Kirkpatrick–Baez (KB) configuration, is deployed for focusing. Bruker ASC (Bergisch-Gladbach, Germany) supplied the KB mirror assembly. Specifications are reported in Table 1 ▸. The KB mirrors generate a minimum focus of 30 µm (H) × 24 µm (V) (FWHM) at the sample position. X-ray intensities for different energies and beam sizes under different focusing/collimating conditions are reported in Fig. 2(*a*) ▸ and in Table S2 of the supporting information. The bimorph mirrors enable the defocusing of the beam both in the horizontal and/or the vertical direction on a timescale of minutes (Fig. 2*b* ▸) up to a beam size of 150 µm (H) and 70 µm (V), without significant loss of photon flux (see the video recording in real time of vertical defocusing in the supporting information). The estimated beam divergence for the 30 µm (H) × 24 µm (V) (FWHM) beam focused at the sample position is predicted to be 230 µrad (H) × 150 µrad (V) (FWHM) (equivalent to ∼0.01°), decreasing to 190 µrad (H) × 110 µrad (V) when the beam is focused at 1 m downstream of the sample position (Fig. S2). The beam profile is close to Gaussian when focused (Fig. 2*c* ▸), while when defocused it shows a characteristic structure with horizontal striations (Fig. 2*d* ▸), as predicted by X-ray tracing (Fig. S2), due to the mismatch between the small vertical beam emittance and the slope errors of the vertical focusing mirror remaining after adaptive optimization. Here, it should be noted that the striated beam profile does not impede the collection of high-quality diffraction data.

### 2.5. Slit systems   

The shape and size of the white beam is defined by a set of water-cooled white-beam slits in the front-end as provided by DESY (Schulte-Schrepping *et al.*, 2013 ▸). Secondary slits at the height of the fixed-exit monochromator suppress the background scatter produced in the DCM and are adjusted to the acceptance of the first of the deflecting mirrors. Two pairs of slits (CINEL, Vigonza, Italy) with a positional reproducibility of 1 µm, one after the HDM and one before the KB system, serve as collimators and define the effectively used surface of the KB mirrors.

### 2.6. Beam monitors   

A permanent fluorescence beam imaging and intensity-monitoring device, harnessing the light emitted from the CVD diamond window (DESY-EMBL development), has been installed immediately upstream of the DCM for alignment of the front-end aperture and white-beam slits.

Removable fluorescence beam imaging screens are deployed after: (*a*) the DCM for its alignment as well as for the source position/shape characterization and diagnostics using the monochromatic beam; (*b*) the HDM pair as a first control of beam deflection; (*c*) the KB system, where knowledge of the beam position is required for alignment of the KB system with respect to the optical axis.

Beam intensity and position monitors are installed at each slit system and after the KB mirrors, and, together with a piezo actuator driven feedback, are used for detecting drifts. Two intensity monitors in the beam-conditioning unit (see below) can be used for precise exposure dose determination.

### 2.7. Diamond windows   

There are currently two CVD diamond windows (60 µm thickness, 5 mm diameter; Diamond Materials GmbH, Freiburg, Germany) installed in the beamline. The first one (36.5 m from the source) is used to separate the front-end vacuum section from the beamline optics, while the second one (56.25 m from the source) separates the vacuum of the beam-conditioning unit from the rest of beamline optics.

### 2.8. Experimental table   

The five-degrees-of-freedom granite experimental table [1.2 m (length, L) × 1.0 m (width, W)], an in-house design and production, supports the beam-conditioning unit, the MD2 and the fluorescence detector.

**Figure 3:**
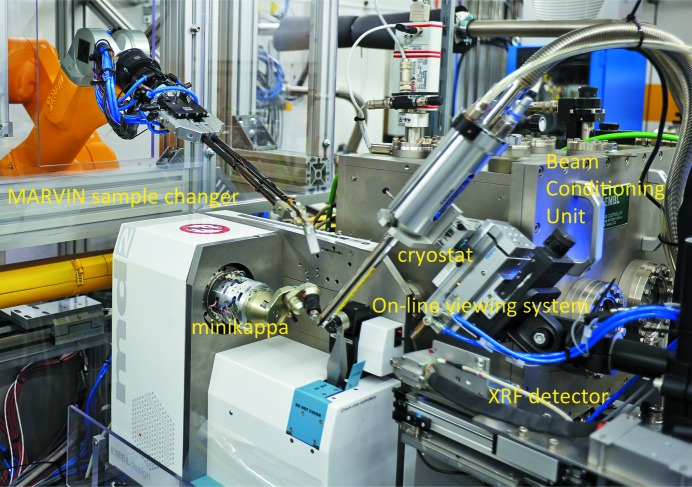
P13 sample environment around the MD2 goniometer with the MARVIN sample changer on the left and the BCU at the back.

The beam-conditioning unit (BCU) is an in-house development of a self-contained in-vacuum unit to collimate, attenuate, characterize and monitor the photon beam. It has a modular design and can be adapted to tight geometrical restrictions. The vessel (Fig. 3 ▸) has a size of 510 mm (L) × 350 mm (W) × 385 mm (H) and houses two fine slits (horizontal and vertical), based on translations by attocube systems AG (München, Germany) with nanometer resolution, a fast shutter module (CEDRAT Technologies, Meylan, France), an attenuator module with an option for 1000 filter combinations and two monitoring modules with a beam intensity monitoring and beam viewing options. The two sets of slits in the BCU (see below) define the optical axis of the experimental table. When a defocused beam is used, the last set of slits serves as a variable beam-defining aperture (30–200 µm) with sub-micrometer precision.

An MD2 diffractometer (Cipriani *et al.*, 2007 ▸) with a mini-κ goniometer head MK3 (ARINAX, Moirans, France) (Figs. 3 ▸ and S5) is installed as the diffractometer. The measured ω-axis sphere-of-confusion (SOC) is 0.9 µm peak-to-peak diameter without the MK3 installed. With the MK3 installed, the SOC is 2.4 µm at κ = 0° with a slight increase (<0.1 µm) for κ = 60° (Fig. S4). The continuous shutterless helical (four-dimensional) scanning capability (Flot *et al.*, 2010 ▸; de Sanctis *et al.*, 2012 ▸) is fully implemented.

A set of fixed-diameter circular apertures at 30 mm distance from the sample position are used either as scatter trap, cleaning or beam-defining aperture depending on the focusing/defocusing conditions. The standard set is a five-hole beam-defining aperture such that the X-ray beam can be shaped to 15, 30, 50, 70 or 100 µm-diameter sizes (Fig. 2*e* ▸); the second set that can be optionally mounted features 50, 10 and 5 µm-diameter apertures and is used to provide mini-beam conditions (Fischetti *et al.*, 2009 ▸). A further scatter trap aperture at 8 mm from the sample position is part of a capillary-backstop assembly (Fig. S5).

A retractable Amptek XR-100SDD fluorescence detector (Amptek, Inc., Bedford, MA, USA) is installed for X-ray absorption-edge scans for MAD experiments and for elemental analysis (Fig. 3 ▸).

### 2.9. Sample changer and cryogenics   

A MARVIN sample changer system (EMBL Hamburg in-house design) is available on P13 (Fig. 3 ▸), with a storage dewar capacity of 16 EMBL/ESRF pucks for SPINE standard pins and vials (Cipriani *et al.*, 2006 ▸), providing a total capacity of 160 samples. A six-axis (STÄUBLI, Pfäffikon, Switzerland) robot is mounted overhead above the storage dewar providing simple access to the samples and reducing the footprint of the system. The crystal gripping mechanism consists of two pneumatic cryogenic tongs mounted at an angle of 45° on carbon rods holding the sample (the cap and the LN2-filled vial). A mounting/dismounting/mounting-next-sample cycle can be performed in less than 60 s. The entire robot arm and the cryo-dewar are installed in a safety enclosure which also serves to maintain a low-humidity atmosphere (dew point < −25°C) minimizing ice formation in the storage dewar. On the diffractometer, the crystals are kept at cryogenic temperatures during data collection by an 800 series Cryostream open flow cryogenic gas cooler (Oxford Cryosystems, Oxford, UK).

### 2.10. Detector and detector table   

**Figure 4:**
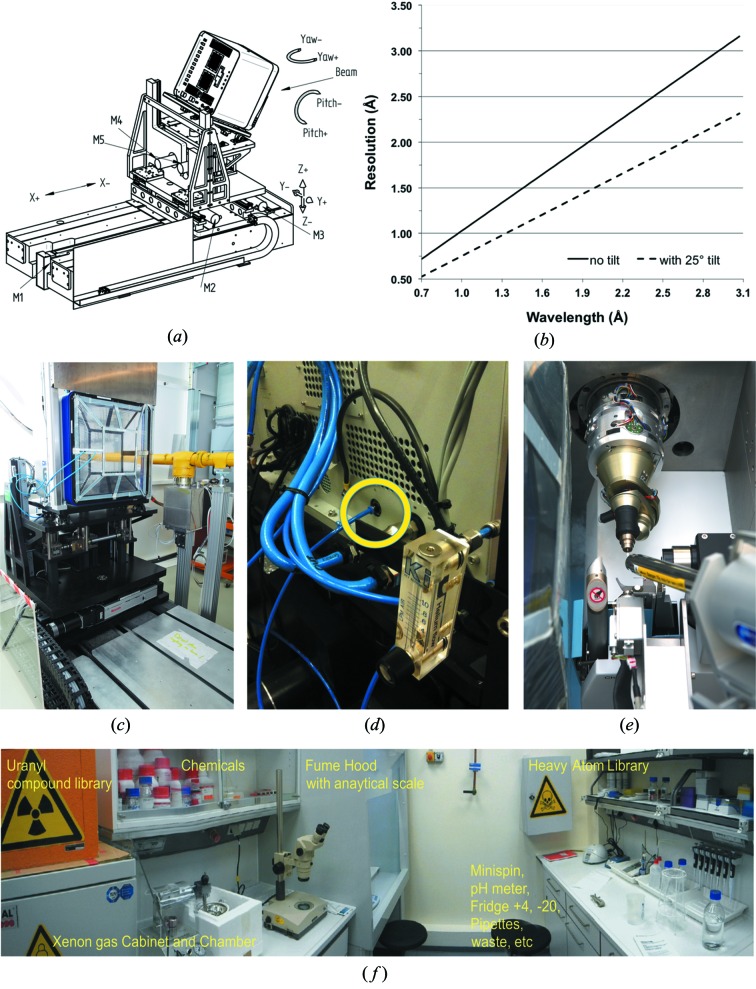
P13 ancillary facilities. (*a*) Drawing of the detector table with vertical 2Θ-arm. (*b*) Diffraction resolution *versus* energy at the edge of the PILATUS 6M-F at 2Θ = 0° (full line) and at 2Θ = 25° (dashed line). (*c*) PILATUS 6M-F equipped with the helium cone assembly. (*d*) Back panel of the DECTRIS PILATUS 6M-F with the standard N_2_ gas inlet highlighted, used also to vent the complete detector with helium gas. (*e*) Detail of the sample position with helium cone assembly on; the post-sample air gap is reduced to 30 mm at the closest sample-to-detector distance of 135 mm. (*f*) EMBL derivative laboratory (DLab) located about 15 m away from the beamline.

The standard detector on P13 is a PILATUS 6M-F (DECTRIS AG, Baden, Switzerland), with 450 µm sensor thickness and custom calibration tables for low energies (Julien *et al.*, 2011 ▸; Marchal & Wagner, 2011 ▸). The detector translation stage (in-house design) offers five degrees of freedom (vertical and horizontal translation, roll, pitch and yaw) (Fig. 4*a* ▸) to ensure detector centring on the direct beam upon the change in the incident beam inclination (after adjustments of the KB mirrors), a large vertical 2Θ offset (up to 25°) and a crystal-to-detector distance adjustable between 135 mm and 1.5 m.

### 2.11. Instrument control   

The control of monochromator, mirrors, robotic sample changer and minor components is carried out by master units containing electronics modules for devices such as motors, encoders and intensity monitors (Ristau *et al.*, 2014 ▸). The modules are interconnected through a real-time Ethernet-based field bus (EtherCAT, Beckhoff™, Verl, Germany) (Pazos *et al.*, 2008 ▸), which allows for simple and fast synchronization of processes inside an individual instrument and between different instruments. The control PCs are compact and are mounted inside the optical and experimental hutches in small units next to the respective beamline components (Fig. S6), thereby reducing cable length and consequently improving the electromagnetic compatibility.

Most of the low-level instrument control (motion control, analogue and digital I/O signal processing, cryogenic and vacuum control system, instrument and personnel safety with exception of the radiation safety interlock system provided by DESY) is implemented in programmable logic controllers (PLC) running on embedded PCs that are part of the master units. Low- and high-level control layers are connected by the TINE control system developed by DESY (Bacher, 2007 ▸). Instrument functionalities can be accessed through TINE servers from different clients for beamline staff and for user operation.

### 2.12. Data acquisition and analysis   

The data acquisition software at P13 is *MxCuBE* (Gabadinho *et al.*, 2010 ▸), developed at ESRF (Grenoble, France), and adapted to the environment of the PETRA III MX beamlines. It supports standard crystallographic data collection protocols, fluorescence scan acquisition and sample changer operation. Logging into *MxCuBE* activates the electronic data collection notebook services provided within *ISPyB* (Delagenière *et al.*, 2011 ▸).

Data management foresees simultaneous storing of two copies of collected data on the PPU on a secondary storage system consisting of two network storage servers (DELL, Frankfurt, Germany) with a total capacity of 300 TB shared between beamlines P12, P13 and P14. The interconnection between storage servers is provided by InfiniBand high-speed data transfer lines. Data transport to external disks is possible *via* USB3 ports.

Data processing is performed with a parallelized version of *XDS* (Kabsch, 2010 ▸) running on a 40-core Pilatus Processing Unit (PPU) XL (DECTRIS AG, Baden, Switzerland). Standard crystallographic software including *CCP4* (Winn *et al.*, 2011 ▸), *PHENIX* (Adams *et al.*, 2010 ▸), *SHELX* (Sheldrick, 2008 ▸) and *HKL2MAP* (Pape & Schneider, 2004 ▸) is available on dedicated graphics and compute servers for structure solution.

### 2.13. Control hutches and user laboratory   

The experimental hutch is directly accessible from the control area, which offers a laboratory bench for small-scale sample preparation, fast data processing, computing for structure solution and refinement, and data back-up. An additional room for data processing and back-up is available in the vicinity of the beamline. For larger-scale sample preparation, a separate user laboratory is available.

## 3. Ancillary facilities   

### 3.1. Data collection at energies below 6 keV   

A number of measures have been introduced to facilitate the collection of anomalous diffraction data at energies below 6 keV. Low scattering background and high photon flux is achieved by an all in-vacuum design all the way to a distance of 50 mm from the sample. The total thickness of the two diamond windows present in the beamline is 120 µm resulting in a transmission of about 20–25% at 4 keV. Higher harmonics are effectively removed by the double-bounce horizontal deflecting mirrors with bare silica, which have a theoretical transmission of 10^−3^ at 12 keV where the highest contribution of the third-harmonic is present (Fig. 1*e* ▸). Potential beam hardening effects, arising from the CVD windows, are therefore strongly reduced. It should be noted that the KB mirrors also comprise bare silica and Rh-coated stripes; however, experience has shown that the use of the silica stripe is not required for further harmonic rejection. A scatter guard and a metal capillary are placed immediately upstream of the sample position (Fig. S5) to minimize the in-air background around the sample.

The PILATUS 6M-F custom calibration tables for low energies (Julien *et al.*, 2011 ▸; Marchal & Wagner, 2011 ▸) enable accurate and low-noise diffraction data collection in the softer X-ray regime.

For 4–6 keV data collections, a custom-made helium cone (fully compatible with the 2Θ-angle detector stage) is fitted to the detector. The assembly of the helium cone and the housing of the PILATUS detector are flushed with a constant helium flow (Figs. 4*c*, 4*d* ▸). For the closest crystal-to-detector distance (135 mm), the helium cone allows to keep 105 mm of the direct X-ray path from the sample to the detector surface in helium atmosphere. In this configuration, the sample can still be kept at 100 K using the standard nitro­gen cryostream (Fig. 4*e* ▸) and operation of the MARVIN sample changer is not affected. Equally, the use of the mini-κ goniostat is possible for Ω angular ranges between 0 and 180° with the helium cone mounted.

**Figure 5:**
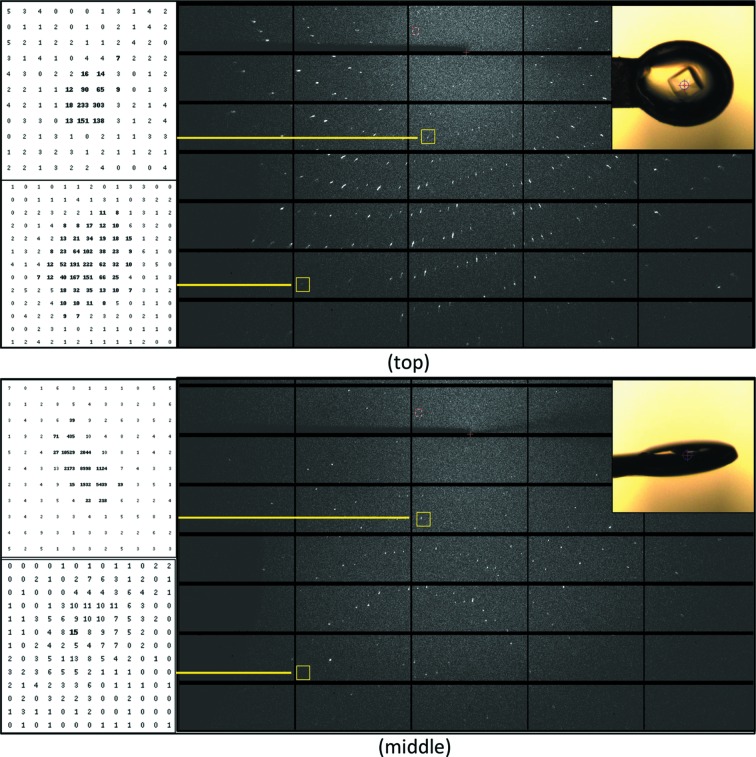
Diffraction images acquired at λ = 2.75 Å (4.5 keV). 1.0° rotation images with an exposure time of 1 s were collected with 3.8 × 10^10^ photons s^−1^ in a beam defocused to 50 µm in diameter at 135 mm crystal-to-detector distance on a crystal of lysozyme (120 µm × 120 µm × 80 µm in size) mounted in a litho-loop (MiTeGEN, Ithaca, USA). Images were displayed with *ADXV* (Arvai, 2015 ▸) using identical contrast levels (−1/+10) with inverted colours. Diffraction images were taken in face-on (top) and edge-on (bottom) orientation of the mounting loop.

The overall endogenous background of P13 at 4.5 keV X-ray energy was measured by inspection of diffraction images of a lysozyme crystal (Fig. 5 ▸) collected with the helium cone in place. The background surrounding the Bragg spots reduces from an average of 1.7 ± 0.1 photons pixel^−1^ s^−1^ at low diffraction angles to 0.13 ± 0.03 photons pixel^−1^ s^−1^ counts pixel^−1^ at high diffraction angles. Exposing the crystal and the mount through their longest dimension marginally alters the background to 1.5 ± 0.1 photons pixel^−1^ s^−1^ at low angles and to 0.19 ± 0.04 photons pixel^−1^ s^−1^ at high angles.

### 3.2. Derivatization laboratory   

A dedicated laboratory ‘DLab’ for handling of heavy atoms and for preparation of solutions for protein derivatization (Fig. 4*f* ▸) is located in the vicinity of the beamline. Currently, the DLab holds a library of more than 150 compounds containing 44 different elements including lanthanide cage compounds (Pompidor *et al.*, 2010 ▸; Talon *et al.*, 2011 ▸) and uranyl derivatives (Liu *et al.*, 2001 ▸). A xenon chamber (Hampton Research, Aliso Viejo, USA) allows preparation of xenon and krypton derivatives for phasing with softer X-rays (Olczak *et al.*, 2003 ▸).

## 4. Facility access   

Access to the EMBL Hamburg beamlines is available to academic research groups, and is prioritized by the Project Evaluation Committee on scientific grounds only. Proposals for beamline access can be submitted to http://smis.embl-hamburg.de. Access for industrial users can be accepted under a commercial agreement. Remote data collection is available.

## 5. Highlights   

At the time of writing, P13 has been accredited with more than 100 Protein Data Bank depositions. Highlights from the available publications include the elucidation of the structural basis of the proinflammatory signalling complex mediated by TSLP (Verstraete *et al.*, 2014 ▸); the crystal structure of the eukaryotic translation initiation factor eIF5B (399–852) from *Saccharomyces cerevisiae* (Kuhle & Ficner, 2014 ▸); the structure and thermodynamics of inhibition of *Sporosarcina pasteurii* urease (Benini *et al.*, 2014 ▸); crystallographic structures of an active Spiegelmer, NOX-D20, bound to its physiological targets, mouse C5a and C5a-desArg (Yatime *et al.*, 2015 ▸). Full MAD and Se-SAD capability are implemented as shown by the crystal structure of *Geobacillus thermoglucosidasius* GH family 52 xylosidase (Espina *et al.*, 2014 ▸) solved with Se-SAD data, or by the in-house structure solution of glucose isomerase with a fluorescence scan around the Pb *L*_III_ edge at 13050 eV (data not shown).

With respect to ultra-high-resolution data collection, the crystal structure of the sunflower trypsin inhibitor 1 (SFTI-1) (Karna *et al.*, 2015 ▸) and the structures of open and closed states of *Candida antarctica* lipase B (Stauch *et al.*, 2015 ▸) were determined at 0.91 Å resolution using X-rays at λ = 1.00 Å (12.4 keV) and λ = 0.826 Å (15.0 keV), respectively.

The close interaction of the beamline with the facility for the characterization, preparation and crystallization of samples (SPC) (Boivin *et al.*, 2016 ▸) facilitated the determination of the crystal structure and function of the first desulfinase with an acyl-CoA de­hydrogenase fold (Schürmann *et al.*, 2015 ▸) and of the levansucrase from *Erwinia amylovora* (Wuerges *et al.*, 2015 ▸), the structural elucidation of the bis­pecificity of A domains as a basis for activating non-natural amino acids (Kaljunen *et al.*, 2015 ▸), and the structural elucidation of 1,4 benzo­quinone inhibition of *Sporosarcina pasteurii* urease (Mazzei *et al.*, 2016 ▸).

### 5.1. Cdc23^Nterm^   

The crystal structure determination of Cdc23^Nterm^, a subunit of the multimeric anaphase-promoting complex (APC) (Cianci *et al.*, 2016 ▸), was solved by sulfur SAD phasing at λ = 2.69 Å (4.6 keV). At this wavelength, the crystal form of Cdc23^Nterm^ studied, containing two molecules of 282 amino acid residues including six cysteines and five me­thio­nines in the asymmetric unit (65.4 kDa, 12 cysteines and 10 me­thio­nines in total), has an expected Bijvoet ratio 〈|*F*_anom_|〉/〈*F*〉 of 2.2% [compared with 0.8% at λ = 1.54 Å (8.0 keV)]. Selectively illuminating two separate portions of the same crystal with an X-ray beam of 50 µm diameter allowed macroscopic crystal twinning to be overcome. The initial experimental phases determined by S-SAD allowed automatic tracing of the dimer at 3.1 Å resolution. Subsequent refinement resulted in a model with *R*_factor_ = 18.7% and *R*_free_ = 25.9%. Increasing the Bijvoet ratio reduced the demands in terms of multiplicity and in crystal supply required to achieve successful phasing. This was of particular importance as only one crystal of high quality was found.

### 5.2. S-SAD phasing of Zn-free insulin at 4 keV   

**Figure 6:**
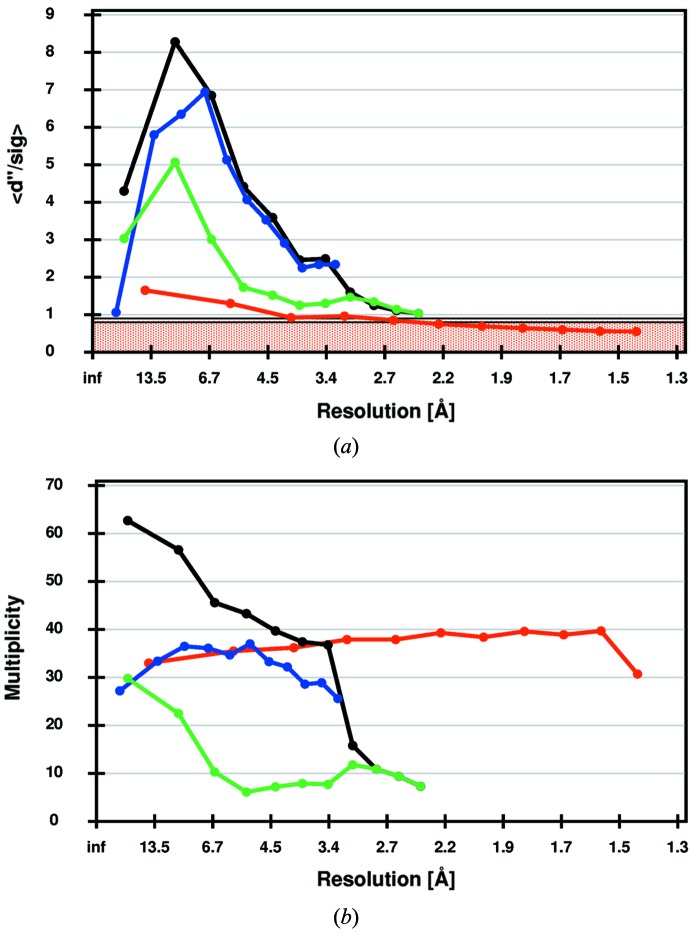
Data quality statistics for crystals of Zn-free insulin as calculated by *SHELXC*. (*a*) Anomalous signal-to-noise *d*′′/sig as a function of resolution. Statistics for data collected at 13 keV are shown in red, for data collected at 4 keV without and with 2Θ-offset in blue and green, respectively, for the combination of the two 4 keV data sets in black. (*b*) Multiplicity as a function of resolution. Colour codes are the same as in panel (*a*).

To demonstrate the feasibility of anomalous data collection at an energy of 4.0 keV, data were collected on a crystal of Zn-free insulin (Faust *et al.*, 2008 ▸) with a beam defocused to 70 µm in diameter using the PILATUS 6M-F detector with the helium cone installed. Two 360° rotation data sets were collected with the 2Θ arm set to 0° and 25°, respectively. A third 360° dataset was collected at an energy of 13.0 keV for phase extension to high resolution (Table S3). For both low-energy data sets, the anomalous signal-to-noise indicates that anomalous differences of high quality were measured despite the lower multiplicity of the data set collected with the 2Θ offset (Fig. 6 ▸).

In terms of phasing the structure, the number of residues traced by *SHELXE* (Sheldrick, 2008 ▸) in the first electron density obtained by *SHELXE*, *N*_traced_, was used as a metric. The respective electron density was derived by combining the unrefined solved substructure, the anomalous differences extracted by *SHELXC*, and 20 cycles of density modification against the 1.4 Å data collected at 13.0 keV. While the structure could actually be solved using any of the two low-energy datasets by themselves or in combination, the data collected at 4 keV with the 2Θ offset (*N*_traced_ = 45) are more useful for phasing than the data collected to a geometrically limited resolution of 3.14 Å at 2Θ = 0° (*N*_traced_ = 26). The best result was obtained by using a combination of the two data sets collected at 4 keV (*N*_traced_ = 49) indicating that the data sets collected at different 2Θ-settings are compatible.

## 6. Discussion and conclusions   

P13 translates the high brilliance of the radiation of PETRA III into a small tunable beam with low divergence at the sample position. The beam can be used for high-resolution data collection on crystals with large unit-cell dimensions, phasing applications exploiting X-ray energies between 4 and 17.5 keV, and high-resolution data collection at high energies. With the instruments in place, it should be possible to resolve diffraction from crystals with unit-cell parameters of 1000 Å to a resolution of 3.0 Å resolution (assuming the PILATUS 6M detector to be placed at a distance of 700 mm from the sample and a spot separation of 4 pixels). Based on the high photon flux and the high detector frame rates, typical data collection times for cryogenically cooled crystals are of the order of a few minutes. In addition to the capabilities geared towards challenging crystallographic applications, the possibility of using relatively large tunable beams with dimensions up to 150 µm with high flux in combination with automatic mounting and remote access allows for applications in high-throughput crystal screening and/or high-throughput data collection.

The beam size can be tailored to the size of the crystal by adjusting the curvature of the bimorph KB mirrors, an operation that can be performed within a few minutes with excellent reproducibility, and by introducing apertures of different diameters into the beam. Low-energy (<6 keV) data collections are possible and yield excellent phases as shown by structure determination of Cdc23^Nterm^ (Cianci *et al.*, 2016 ▸). With the use of a helium cone, the immediate vicinity of the sample remains accessible as to facilitate standard use of the mini-κ goniostat and of the robotic sample mounter.

Overall, the combination of the adjustable focal properties and robust wide-range energy-tunability of the beamline allow optimal conditions to be chosen for both native and anomalous data collections on a wide range of crystals.

## 7. Related literature   

The following references are mentioned in the supporting information: Sanishvili *et al.* (2008 ▸); Wikoff *et al.* (2000 ▸).

## Supplementary Material

Supporting Tables S1 to S3; Figs. S1 to S6.. DOI: 10.1107/S1600577516016465/ig5037sup1.pdf

Real-tme movie showing vertical defocusing. DOI: 10.1107/S1600577516016465/ig5037sup2.avi

## Figures and Tables

**Table 1:** PETRA III and EMBL P13 beamline details

Storage ring	PETRA III, DESY, Germany
Circumference	2304 m
Particle energy	6 GeV
Particle current (planned)	100 (200) mA
Horizontal emittance	1 nm rad
	
Beamline name	P13
Source type	U29-2, 2 m
Monochromator (36.8)†	Oxford-FMB, UK; Si(111) or Si(311); both crystals LN2 cooled, fixed exit
First deflecting mirror (38.7)†	3.8 mrad, silica substrate + Rh coating, 400 mm optical surface length [0.32 µrad]
Second deflecting mirror (39.7)†	3.8 mrad, silica substrate + Rh coating, 400 mm optical surface length [0.29 µrad]
Horizontal focusing mirror (52.75)†	3.8 mrad, silica substrate + Rh coating, 400 mm optical surface length [0.39 µrad with adaptive mirror corrections]
Vertical focusing mirror (53.75)†	3.8 mrad, silica substrate + Rh coating, 250 mm optical surface length [0.44 µrad with adaptive mirror corrections]
Energy range (keV)	4–17.5
Wavelength range (Å)	3.1–0.7
Beam size [focused, defocused, H × V (µm)] FWHM (57.25)†	30 × 24, 150 × 70
Beam diameter (collimated, typical) (µm)	5, 10, 15, 30, 50, 70, 100
Beam divergence (H × V) (mrad)	0.23 × 0.15
Goniometer	ARINAX MD2, horizontal single axis, with mini-κ goniometer option
Cryo capability	Cryostream 800 series (Oxford CryoSystems, Oxford, UK)
Sample mounting	Manual/MARVIN Sample Changer, capacity of 16+1 EMBL/ESRF pucks with SPINE standard pins and vials
Detector model	DECTRIS PILATUS 6M-F with custom calibration table for energies 4–20 keV
2θ capabilities	Yes, vertical 25°

† Distance in meters from the source.
