# Supplementary material for: P13, the EMBL macromolecular crystallography beamline at the low-emittance PETRA III ring for high- and low-energy phasing with variable beam focusing
Source: J Synchrotron Radiat. 2017 Jan 1;24(Pt 1):323–32. doi: 10.1107/S1600577516016465 (PMC5182027; doi:10.1107/S1600577516016465)
Supplement: Supplementary file 1 [file s-24-00323-sup1.pdf]

JOURNAL OF SYNCHROTRON RADIATION

Volume 24 (2017)

Supporting information for article:

P13, the EMBL macromolecular crystallography beamline at the low emittance PETRA III ring for high and low energy phasing with variable beam focusing

Michele Cianci, Johanna Kallio, Guillaume Pompidor, Gleb Bourenkov, Manfred Rossle, Stefan Fiedler and Thomas R. Schneider

Table S1 EMBL P13 construction timeline.

Date	Step
13th December 2010	First monochromatic beam
13th December 2011	First data collection
28th August 2012	First unknown protein solved by S-SAD
17th November 2012	First friendly external user group
13th February 2013	Begin of user operation
29th November 2013	User operation with MARVIN sample changer
31st January 2014	First user to screen and collect data from 200 samples
3rd February 2014	Begin of a fifteen months shutdown

Table S2 Measured beam size and intensity for different energies and focusing/collimating conditions at P13. Intensity is reported as [photons s^{-1} $(100mA)^{-1}$].

Energy (keV)	Nominal beam size 30 × 24 (H × V) μm^2	Beam-defining aperture diameter 10 μm	Beam-defining aperture diameter 5 μm
17.5	$5.4 \times 10^{+11}$	$5.9 \times 10^{+10}$	$1.5 \times 10^{+10}$
15	$4.2 \times 10^{+12}$	$4.7 \times 10^{+11}$	$1.2 \times 10^{+11}$
13	$4.4 \times 10^{+12}$	$4.8 \times 10^{+11}$	$1.2 \times 10^{+11}$
11	$8 \times 10^{+12}$	$4.1 \times 10^{+11}$	$1.1 \times 10^{+11}$
9	$6.3 \times 10^{+12}$	$6.9 \times 10^{+11}$	$1.8 \times 10^{+11}$
6	$1.6 \times 10^{+12}$	$1.7 \times 10^{+11}$	$4.3 \times 10^{+10}$
4.6	$3.1 \times 10^{+11}$	$3.4 \times 10^{+10}$	$8.7 \times 10^{+09}$

Table S3 Data collection and refinement statistics of data sets collected at EMBL P13 with DECTRIS PILATUS 6M on crystals of Zn-free insulin.

**Data collection**	**4.0 keV**	**13.0 keV**	**4.0 keV - 2Θ**
**crystals size**	200 × 200 × 150 μm³	200 × 200 × 150 μm³	200 × 200 × 150 μm³
Wavelength (Å)	3.099	0.826	3.099
Beam parameters	focused beam with 100 μm ∅ aperture	focused beam with 100 μm ∅ aperture	focused beam with 100 μm ∅ aperture
Attenuation (%)	10	10	10
Exposure time (msec)	40		
Mini-kappa used	no		
Crystal-to-Detector distance (mm)	136	278.96	136
2Θ angle (°)	0	0	25
Oscillation angle (degrees)	0.1		
Number of images	3600		
Space group	*I*2₁3		
(α, β, γ, degrees)	90, 90, 90		
Unit cell (a, b, c, Å)	77.9, 77.9, 77.9	78.9, 78.9, 78.9	77.9, 77.9, 77.9
Resolution range (Å)[1]	55.13 - 3.14 (3.39 – 3.14)	55.79 – 1.43 (1.44 – 1.43)	55.11 – 2.34 (2.42 – 2.34)
Total number of reflections[1]	45562	576586	32446
Unique reflections[1]	1466	15455	3442
Multiplicity[1]	31.1 (26.3)	37.3 (11.3)	9.4 (6.7)
Completeness[1] (%)	99.7 (98.6)	98.8 (75.7)	99.3 (93.7)
R_{merge}[1,2]	7.9 (0.112)	5.5 (157.2)	8.4 (19.3)
R_{merge} low resolution shell	8.4	4.7	7.4
Mean I/s(I)[1]	46.4 (29.9)	32.9 (1.4)	20.5 (7.4)
Mid-Slope of Anomalous Normal Probability	3.698	0.883	1.606

[1]highest resolution bin in parenthesis

[2]Rmerge = $\Sigma_{hkl} \Sigma j \mid Ij - \langle I \rangle \mid / \Sigma_{hkl} \Sigma j\ Ij$ where I is the intensity of a reflection, and <I> is the mean intensity of all symmetry related reflections j.

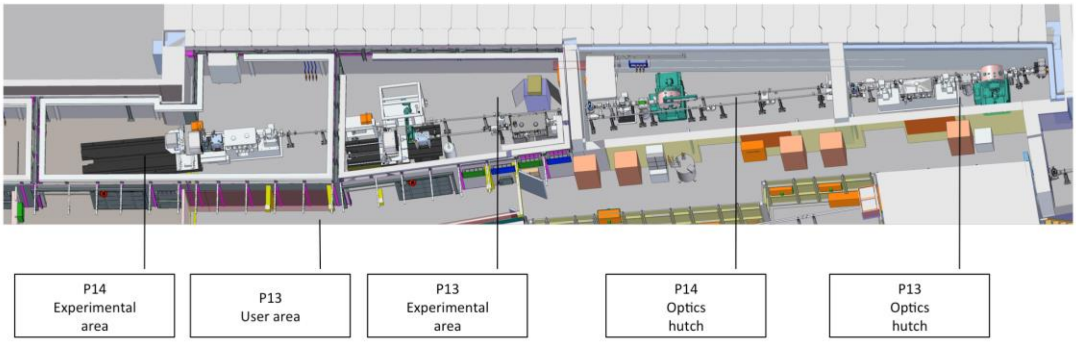

Figure S1 Bird-view of the EMBL P13 and P14 beamlines on sector 9 of the PETRA III storage ring (DESY, Hamburg, DE).

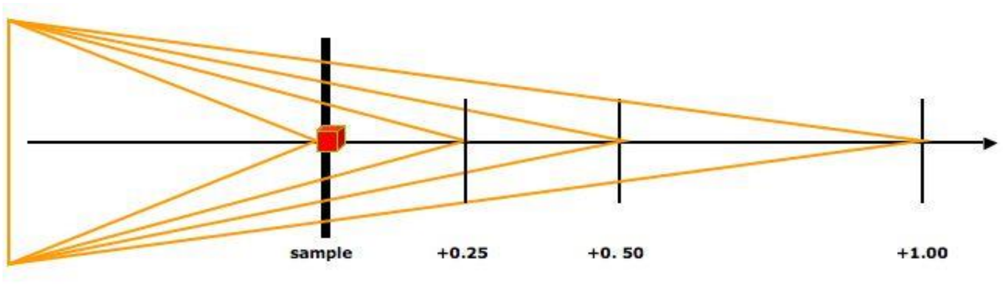

**Focus position (m)**	**Sample**	**+0.25**	**+0.50**	**+1.00**
**Beam size at sample (µm)**	47x23	69x33	110x56	200x112
**Beam divergence (mrad)**	0.23x0.15	0.22x0.14	0.21x0.12	0.19x0.11
**Beam profile at sample**	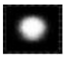	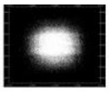	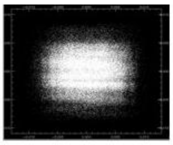	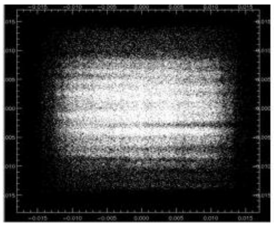

Figure S2 Scheme of various focusing options envisaged for P13 during design. The X-ray tracing was performed with XOP SHADOWGUI version 1.0 Beta4.2.1 (http://www.esrf.fr/computing/scientific/xop) with 250k rays at 12 keV with mirror slope errors of 0.5 µrad. The advantage of matching the beam diameter to the crystal size is the improvement in the signal-to-noise ratio by optimization of the irradiated crystal volume (signal) against irradiated volume of the crystal mount (noise) (Fischetti et al., 2009, Sanishvili et al., 2008). When the beam focus is placed at the sample position, the beam divergence at P13 is in the order of ~ 0.01°. Defocusing the beam will move the focus towards the detector surface and simultaneously will further reduce the divergence – both conditions being of advantage for resolving diffraction spots from large crystallographic unit cells (Wikoff et al., 2000) Nave (1999).

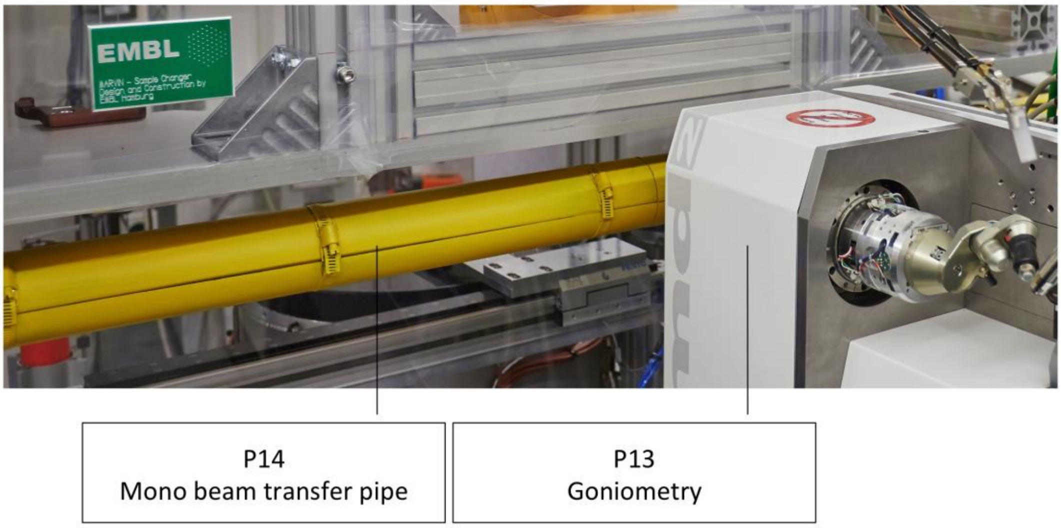

Figure S3 The monochromatic-beam transfer pipe of beamline P14 passing next to the P13 MD2 diffractometer.

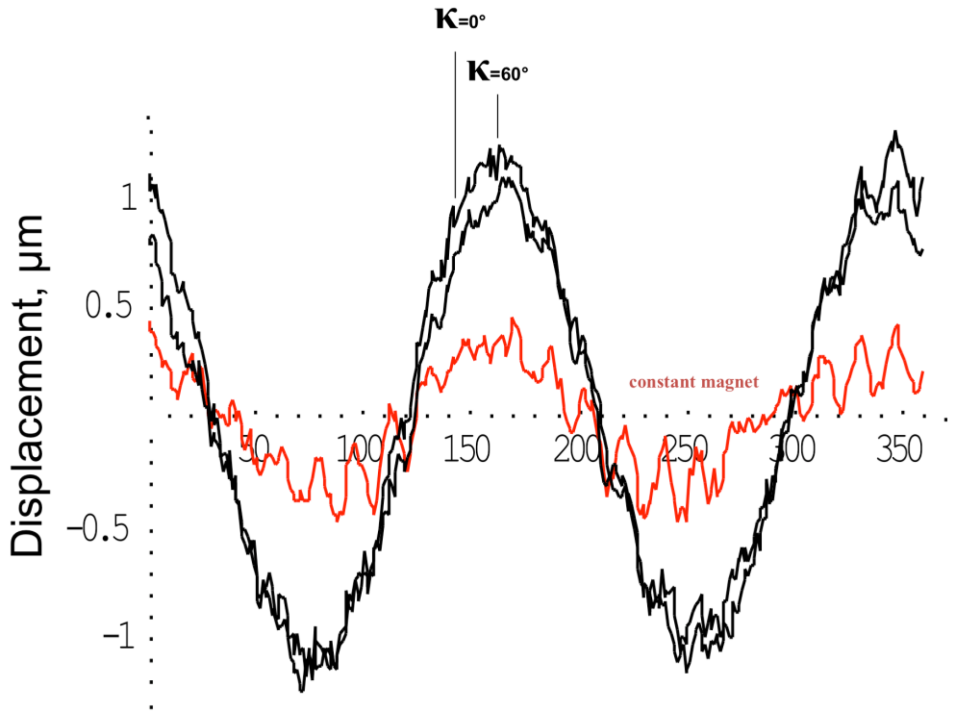

Figure S4 Sphere-of-confusion with miniKappa: 0.7 μm rms (2.4 μm peak-to-peak diameter).

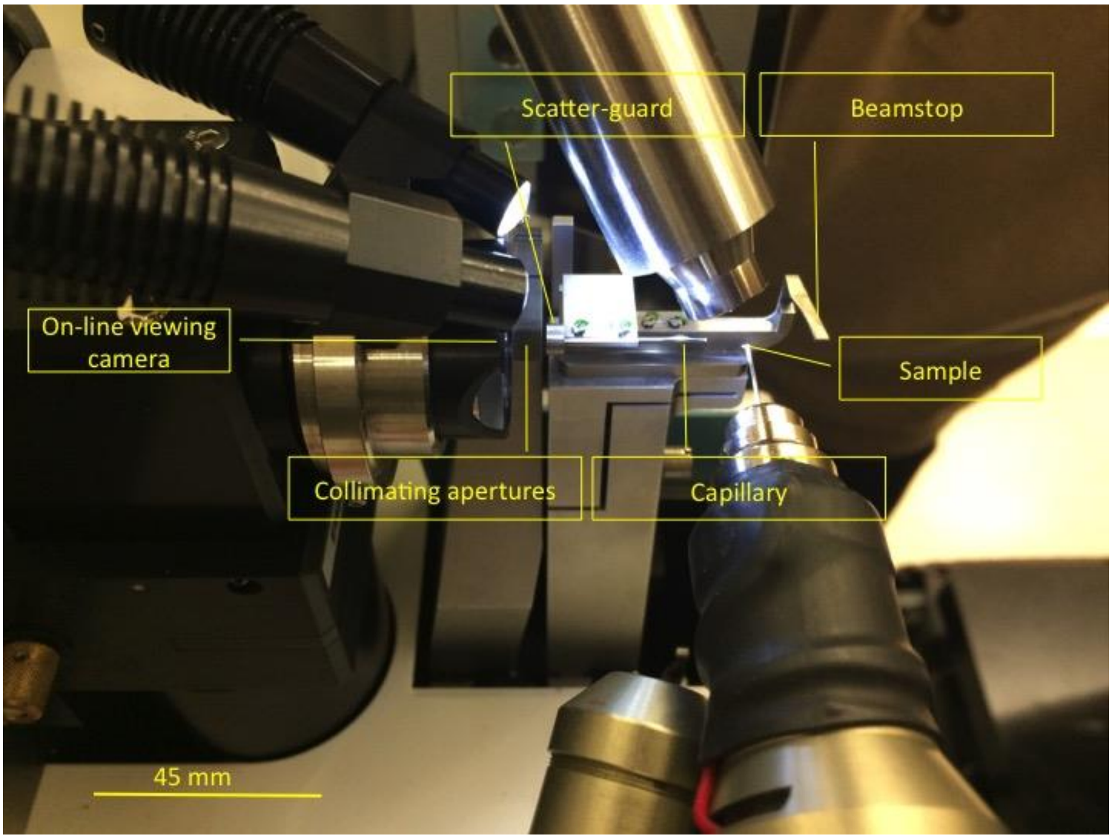

Figure S5 Sample environment at P13.

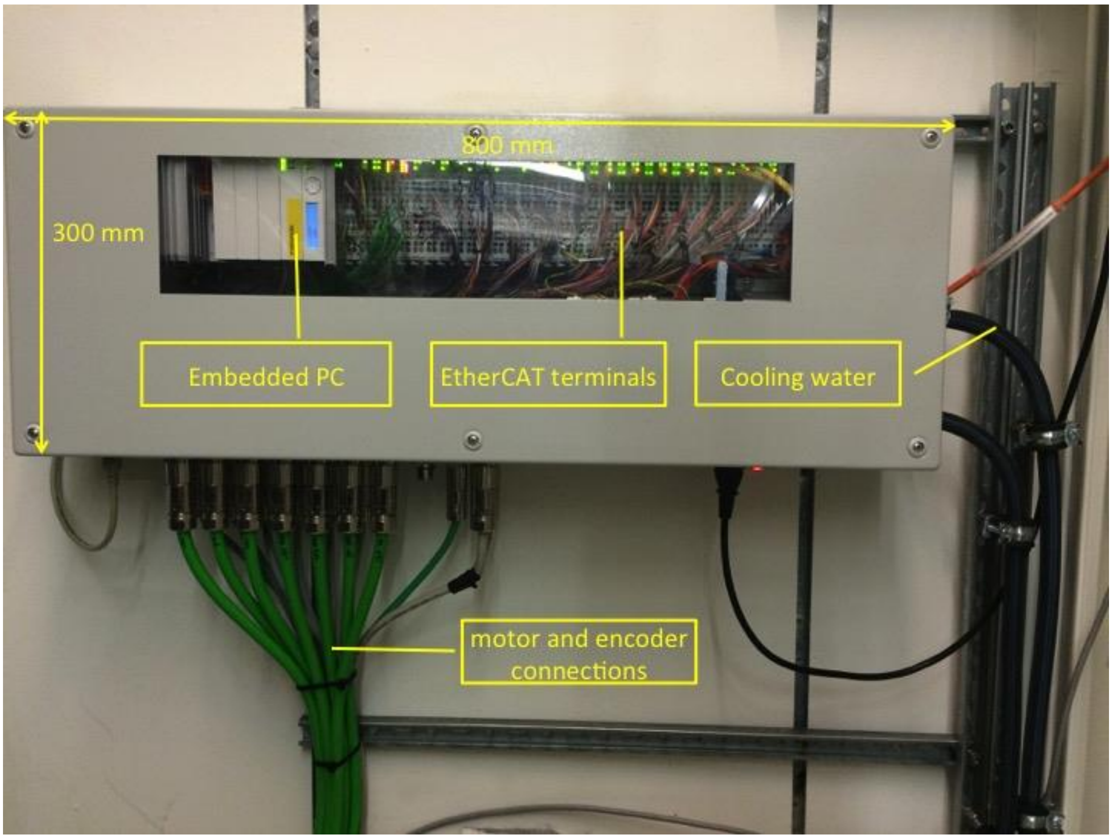

Figure S6 A typical motion control unit (Beckhoff box) at the EMBL beamlines.

References

Fischetti, R. F., Xu, S., Yoder, D. W., Becker, M., Nagarajan, V., Sanishvili, R., Hilgart, M. C., Stepanov, S., Makarov, O. & Smith, J. L. (2009). *J. Synchrotron Rad.* **16**, 217-225.

Sanishvili, R., Nagarajan, V., Yoder, D., Becker, M., Xu, S., Corcoran, S., Akey, D. L., Smith, J. L. & Fischetti, R. F. (2008). *Acta Cryst.* D**64**, 425-435.

Wikoff, W. R., Schildkamp, W. & Johnson, J. E. (2000). *Acta Cryst.* D**56**, 890-893.
